# Enhancing Bangla handwritten character recognition using Vision Transformers, VGG-16, and ResNet-50: a performance analysis

**DOI:** 10.3389/fdata.2025.1682984

**Published:** 2025-11-14

**Authors:** A. H. M. Shahariar Parvez, Md. Samiul Islam, Fahmid Al Farid, Tashida Yeasmin, Md. Monirul Islam, Md. Shafiul Azam, Jia Uddin, Hezerul Abdul Karim

**Affiliations:** 1 Department of Software Engineering, Daffodil International University, Dhaka, Bangladesh; 2 Department of Computer Science and Engineering, State University of Bangladesh, Dhaka, Bangladesh; 3 Centre for Image and Vision Computing (CIVC), COE for Artificial Intelligence, Faculty of Artificial Intelligence and Engineering (FAIE), Multimedia University, Cyberjaya, Selangor, Malaysia; 4 Department of Computer Science and Engineering, Atish Dipankar University, Dhaka, Bangladesh; 5 Department of Information and Communications Engineering, Hankuk University of Foreign Studies, Seoul, Republic of Korea; 6 Department of Computer Science and Engineering, Pabna University of Science and Technology, Pabna, Bangladesh; 7 Artificial Intelligence and Big Data Department, Woosong University, Daejeon, Republic of Korea

**Keywords:** deep learning, Bangla handwritten character recognition, optical character recognition, convolutional neural network, Vision Transformer (ViT), VGG-16, ResNet-50

## Abstract

Bangla Handwritten Character Recognition (BHCR) remains challenging due to complex alphabets, and handwriting variations. In this study, we present a comparative evaluation of three deep learning architectures—Vision Transformer (ViT), VGG-16, and ResNet-50—on the CMATERdb 3.1.2 dataset comprising 24,000 images of 50 basic Bangla characters. Our work highlights the effectiveness of ViT in capturing global context and long-range dependencies, leading to improved generalization. Experimental results show that ViT achieves a state-of-the-art accuracy of 98.26%, outperforming VGG-16 (94.54%) and ResNet-50 (93.12%). We also analyze model behavior, discuss overfitting in CNNs, and provide insights into character-level misclassifications. This study demonstrates the potential of transformer-based architectures for robust BHCR and offers a benchmark for future research.

## 1 Introduction

With over 285 million speakers, most of them in Bangladesh and India, Bengali is one of the most widely spoken languages in the world. Approximately 98% of people in Bangladesh speak it as their first language (Wikipedia, 2024), and it is the official and national language of the country. Bangla has a complex script with fifty alphabets, ten numerals, and numerous compound and unique characters. The intricate forms, widths, styles, and strokes of Bangla handwritten characters, along with similarities between certain characters, make manual identification challenging. Despite its importance, studies on Bangla Optical Handwritten Character Recognition (OHCR), especially for complex characters, remain limited.

In this work, we leverage the Vision Transformer (ViT), a cutting-edge deep learning model for image classification, to address these challenges (Dosovitskiy et al., 2020). OCR converts handwritten text into machine-readable formats and has applications in accessibility, document analysis, digitization, and translation. While state-of-the-art OCR models exist for languages such as English, Mandarin, Spanish, and French, Bangla OCR remains less explored, particularly for compound characters. The complexity of Bangla script demands advanced feature extraction and representation, as some characters differ only by subtle strokes or dots.

Recent deep learning advancements, including VGG16 (Simonyan and Zisserman, 2014), InceptionV3 (Xia et al., 2017), ResNet (He et al., 2016), and ViT, provide powerful tools for addressing these challenges. ViT, based on the Transformer architecture initially developed for natural language processing, captures both local and global image features through self-attention mechanisms, outperforming conventional CNNs in many image recognition tasks.

The proposed method leverages ViT to overcome challenges in character complexity. The input image is divided into patches, which are processed as tokens through multiple Transformer layers. ViT's self-attention effectively captures both local and global patterns, enabling recognition of simple and compound Bangla characters from scanned images.

Our work focuses on Bangla Optical BHCR using ViT and popular CNN architectures (VGG-16 and ResNet-50). Although several datasets exist, we employ the CMATERdb 3.1.2 dataset, a widely used benchmark comprising 24,000 images across 50 classes. Despite being extensively studied, CMATERdb provides a standardized environment to evaluate model performance, facilitating fair comparisons with prior work. Evaluation on additional datasets, such as BanglaLekha or Ekush, is considered for future research.

This study demonstrates that ViT outperforms CNNs on CMATERdb, achieving state-of-the-art accuracy while providing insights into model generalization, regularization, and feature extraction strategies. Our main contributions are:

- A comprehensive review of existing BHCR literature, highlighting challenges and opportunities.
- A detailed comparison of CNN and ViT architectures, emphasizing their suitability for BHCR.
- Extensive experiments and analyses on the CMATERdb dataset using three deep learning models.
- Demonstration that ViT achieves state-of-the-art accuracy of 98.26% on CMATERdb, outperforming standard CNN models.
- Evidence that data augmentation and fine-tuning significantly enhance model performance and robustness, particularly for ViT.
- Analysis of ViT's feature extraction capabilities, showing its ability to capture long-range dependencies and subtle character distinctions that CNNs struggle with.

The remainder of this paper is organized as follows: Section 2 reviews the literature; Section 3 presents the methodology; Section 4 analyzes results and discussion; and Section 5 concludes the paper.

## 2 Literature review

The BHCR has attracted significant research interest, with most studies leveraging deep learning to address challenges such as high inter-class similarity, compound characters, and diverse handwriting styles. Existing works can be broadly categorized into CNN-based methods, transfer learning approaches, lightweight architectures, and early applications of Transformer models.

### 2.1 CNN-based architectures

Traditional CNNs have been the backbone of BHCR. Halder et al. (2023) proposed a CNN tailored for Bangla characters, reporting high accuracy on CMATERdb 3.1.2. Similarly, Hasan et al. (2021) developed an 11-layer CNN, achieving 98.03% accuracy. Ghosh et al. (2021) used MobileNetV1, showing promising results across basic, compound, and numeral characters. These works demonstrate CNNs' strong ability to capture local spatial features. However, they remain prone to overfitting and often struggle with complex compound characters.

### 2.2 Transfer learning approaches

Transfer learning has been explored to leverage pretrained models. Dipu et al. (2021) applied VGG and ResNet-based architectures to the BanglaLekha-Isolated dataset, achieving up to 96.88% accuracy. Ghosh et al. (2020) tested InceptionResNetV2 and DenseNet121 on CMATERdb, achieving 96.99%. While these approaches reduce training cost, they are computationally expensive and often biased toward source-domain features, limiting robustness for Bangla-specific scripts.

### 2.3 Lightweight and custom CNNs

Several studies proposed lightweight CNNs for efficiency. Sayeed et al. (2021) developed BengaliNet, tested across CMATERdb, BanglaLekha, Ekush, and NumtaDB, achieving 96%–99% accuracy. Rabby et al. (2018) introduced BornoNet and EkushNet as compact CNN/DNN models for mixed datasets. Similarly, Saha et al. (2019) proposed BBCNet-15 with 96.40% accuracy. While these models improve efficiency, they still rely heavily on handcrafted architecture design and lack the ability to capture long-range dependencies.

### 2.4 Challenges in BHCR

Despite progress, most CNN-based works face common limitations: (i) difficulty in handling visually similar characters, (ii) limited generalization across different writing styles or datasets, and (iii) reliance on local receptive fields, making global structural dependencies harder to learn. These issues highlight the need for models capable of capturing both local and global features effectively.

### 2.5 Applications of deep learning beyond character recognition

Deep learning has been successfully applied across a wide range of domains beyond character recognition. For instance, fine-tuned AlexNet has been explored for crowd anomaly detection in video frames (Khan et al., 2022b), while ensemble machine learning methods have been used to detect energy theft in smart meters (Kawoosa et al., 2023). Similarly, RNN variants enhanced with hybrid optimization algorithms have improved human motion detection under occlusion (Cheltha et al., 2024), and IoT-assisted systems have utilized deep learning for context-aware fertilizer recommendation (Khan et al., 2022a). These studies highlight the adaptability of machine learning and deep learning approaches across heterogeneous application areas, reinforcing the motivation to apply such techniques for Bangla handwritten character recognition. In contrast to these applications, our study focuses specifically on enhancing Bangla handwritten character recognition using Vision Transformers, VGG-16, and ResNet-50, providing a comparative performance analysis.

### 2.6 Emergence of transformer models

Transformers, particularly Vision Transformers (ViT), have recently shown potential for handwriting recognition beyond Bangla. For instance, Geng et al. (2023) proposed LW-ViT for Chinese handwritten characters, reducing complexity while maintaining high accuracy (95.8%). However, to the best of our knowledge, ViT has not yet been systematically applied to Bangla handwritten characters. This represents a critical research gap.

### 2.7 Positioning of this study

Building on these insights, our work makes two contributions: (1) We apply Vision Transformers to BHCR systematically, directly addressing the limitations of CNNs by leveraging ViT's ability to model long-range dependencies via self-attention. (2) Through comparison with VGG16 and ResNet50, we demonstrate that ViT not only achieves the highest accuracy on CMATERdb 3.1.2 but also exhibits superior generalization, narrowing the gap between training and validation performance.

These contributions position our study as a significant step forward in the field, extending the frontier of Bangla character recognition beyond CNNs and transfer learning into Transformer-based architectures.

## 3 Methodology

**Figure 1:**
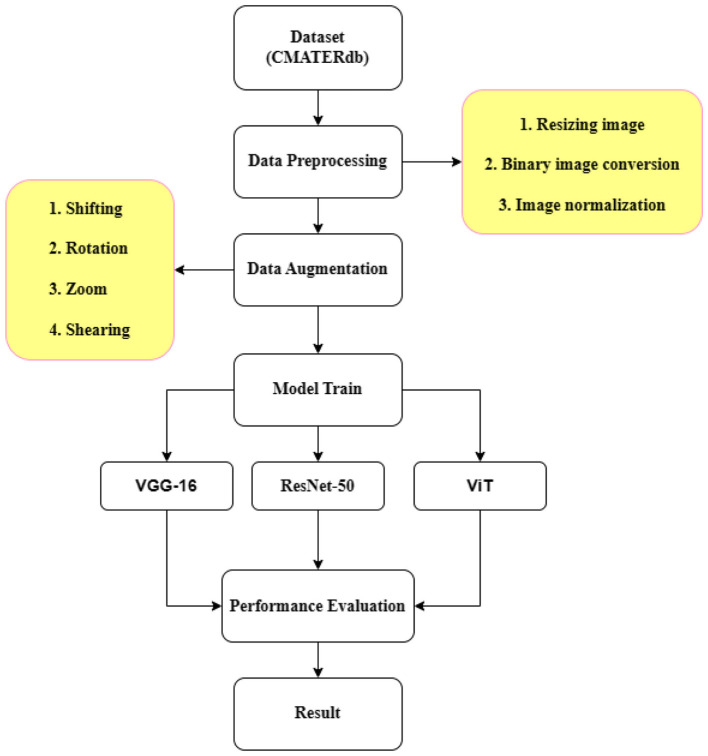
A block diagram of methodology.

Figure 1 provides a visual representation of the entire methodology. The subsequent sections will delve into each of these steps in more detail. The process begins with the CMATERdb 3.1.2 dataset, which is subjected to various preprocessing steps to prepare it for the subsequent stages. The preprocessing and augmentation phase includes several techniques such as shifting, rotation, zooming, and shearing. These techniques help to increase the diversity of the data, thereby improving the robustness of the models. Additionally, the images are resized, converted to binary format, and normalized to ensure uniformity and to facilitate efficient processing. Following data preprocessing and augmentation, we train three different models: VGG16, ResNet50, and ViT. Each of these models has its unique strengths and contributes to the overall performance of our system. After training, we measure the performance of each model. The performance metrics include training and validation accuracy, which provide insights into the effectiveness of each model in recognizing Bangla handwritten characters.

### 3.1 Dataset description

**Figure 2:**
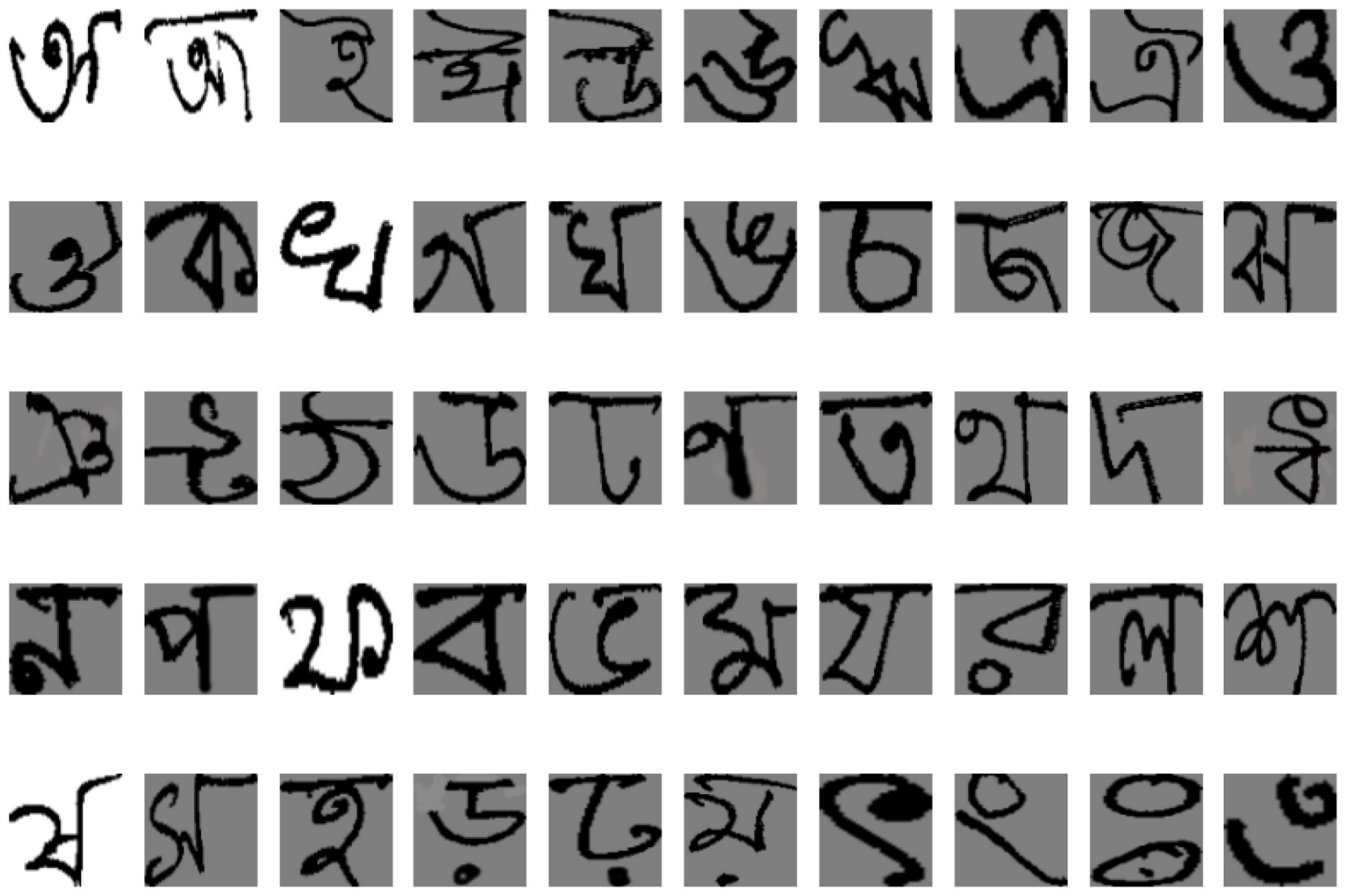
Example of CMATERdb 3.1.2 dataset.

The Center for Microprocessor Applications for Training Education and Research (CMATER) lab at Jadavpur University, India, developed CMATERdb, one of the earliest databases for handwritten Bengali alphabets. Among its versions, CMATERdb 3.1.2 contains 24,000 images of 50 distinct Bengali basic characters, making it suitable for Bangla handwritten character recognition (Sarkar et al., 2012). The dataset is well-balanced, with a sufficient number of samples per class, and has been widely used in prior research, providing a reliable benchmark for model comparison. Figure 2 shows a random image from all 50 classes.

For this study, the dataset was split into training and validation sets using an 80:20 ratio. The split was performed randomly while maintaining stratification across all classes to ensure proportional representation. A fixed random seed was applied for reproducibility. This controlled partitioning allows consistent evaluation of model performance and enables focused analysis of overfitting, regularization, and generalization behavior on small-scale data. Future work may extend this evaluation to larger and more diverse datasets, such as BanglaLekha or Ekush, to further validate model robustness.

### 3.2 Data preprocessing and augmentation

The preparation of the dataset for training involves two key steps: data preprocessing and data augmentation. In this study, all images were resized to 72 × 72 pixels and normalized to the [0,1] range. These steps ensure consistent input dimensions and scale across the dataset.

To increase training data diversity and improve model robustness, we applied standard augmentation techniques including:

- Random rotation (±10°).
- Horizontal and vertical shifts (0.1 fraction of image size).
- Zooming (0.1).
- Shearing (0.1).

**Figure 3:**
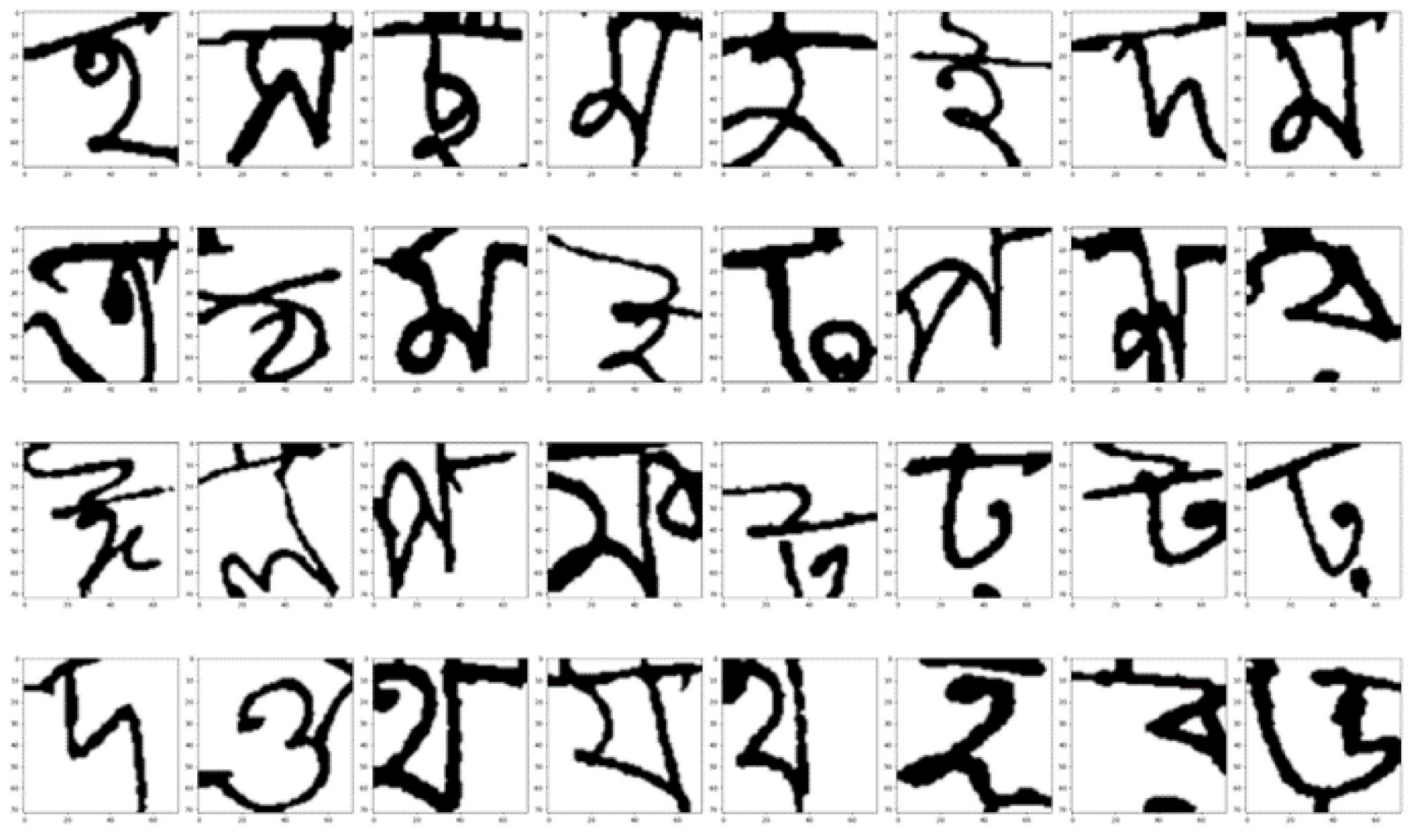
Sample images after preprocessing and augmentation.

The CMATERdb 3.1.2 dataset is largely balanced, with roughly equal samples per class. This helps prevent bias toward specific characters and ensures fair evaluation of model performance across all 50 classes. Figure 3 shows sample images after preprocessing and augmentation. The dataset is largely balanced, with roughly equal samples per class, preventing bias toward specific characters.

### 3.3 Model training and configurations

The BHCR requires extracting complex and varied features from handwritten characters, which traditional machine learning methods with handcrafted features often fail to capture. Deep learning (DL) approaches, including CNNs such as VGG-16 and ResNet-50, and ViT, overcome these limitations by automatically learning hierarchical and global features. CNNs excel at capturing local spatial patterns, with performance influenced by network depth, filter sizes, and hyperparameters such as learning rate, batch size, dropout, and optimizer choice. ViT processes images as sequences of patches and employs self-attention to capture long-range dependencies, with key hyperparameters including patch size, embedding dimension, number of layers and heads, dropout, and learning rate. These DL-based models have shown strong generalization and accuracy for BHCR tasks. The key configurations are summarized below.

- **VGG-16 and ResNet-50:** Adam optimizer, categorical cross-entropy loss, batch size 32, learning rate 0.0001, trained for 100 epochs (VGG-16, ResNet-50), dropout rate 0.5, with early stopping based on validation loss.
- **Vision Transformer (ViT):** Patch size = 6, embedding dimension = 64, 12 transformer layers, 8 attention heads, AdamW optimizer with weight decay 0.01, batch size 32, learning rate 0.0001, trained for 100 epochs.

#### 3.3.1 Training and hyperparameter settings

Table 1 summarizes the training and fine-tuning hyperparameters used for BHCR experiments. VGG-16 and ResNet-50 were initialized with ImageNet-pretrained weights, while ViT was trained from scratch. CNN models employed the Adam optimizer, whereas the Vision Transformer used AdamW to account for decoupled weight decay. Learning rates indicate the initial value for each model, with LR scheduling applied during training to improve convergence. Dropout values are specified per model, with ViT employing separate rates for the encoder (0.1) and the MLP classification head (0.5). The Notes column provides additional model-specific configurations, including the fine-tuned layers for CNNs and the patch size, embedding dimension, number of attention heads, number of Transformer layers, and MLP head units for ViT.

**Table 1:** Training and fine-tuning hyperparameters for BHCR experiments.

**Model**	**Pre-trained**	**Optimizer**	**LR**	**Batch Size**	**Epochs**	**Dropout**	**Notes**
VGG-16	ImageNet	Adam	1e-4	128	100	0.5	Fine-tuned top convolutional blocks
ResNet-50	ImageNet	Adam	1e-4	128	100	0.5	Fine-tuned top residual stages
ViT	Scratch	AdamW	1e-3	128	100	0.1/0.5	Patch size = 6, d = 64, h = 4, L = 8, MLP head = [2,048, 1,024]

CNNs used Adam, ViT used AdamW. Dropout is 0.5 for CNNs, and 0.1 (encoder), 0.5 (MLP head) for ViT.

We selected VGG16, ResNet50, and ViT to provide a comprehensive comparison between classical CNN-based architectures and the recent Transformer-based models for handwritten character recognition. VGG16 and ResNet50 are among the most widely used CNN architectures for image classification and OCR tasks, offering a strong and well-established baseline for BHCR. In contrast, ViT represents a newer class of architectures that leverage self-attention mechanisms to capture both local and global dependencies, making it suitable for handling complex handwritten Bangla characters. By comparing these three architectures, we aim to highlight the strengths and limitations of each model and demonstrate whether ViT can offer a meaningful performance gain over traditional CNNs for BHCR.

## 4 Result analysis and discussion

All models were trained using mini-batch training, with batch sizes as specified in Table 1. Gradients were computed and model parameters updated using the Adam optimizer for VGG-16 and ResNet-50, and AdamW for ViT. Cross-entropy loss was used to measure the discrepancy between predicted and true class labels. Initial learning rates were set according to Table 1, with learning rate scheduling applied during training. The input image size followed the configuration specified for each model in the previous section.

For performance evaluation, accuracy, precision, recall, and F1 score were calculated from the confusion matrix, as defined in Equations 1–4, respectively.


$$\begin{array}{l} Accuracy=\frac{Number\;of\;Correct\;Prediction}{Total\;Number\;of\;Prediction} \end{array}$$ (1)

$$\begin{array}{l} Precision\;=\;\frac{True\;Positive}{True\;Positive\;+\;False\;Positive} \end{array}$$ (2)

$$\begin{array}{l} Recall=\frac{True\;Positive}{True\;Positive\;+\;False\;Negative} \end{array}$$ (3)

$$\begin{array}{l} F-1Score=\frac{2\times Precision\times Recall}{Precision+Recall} \end{array}$$ (4)

**Figure 4:**
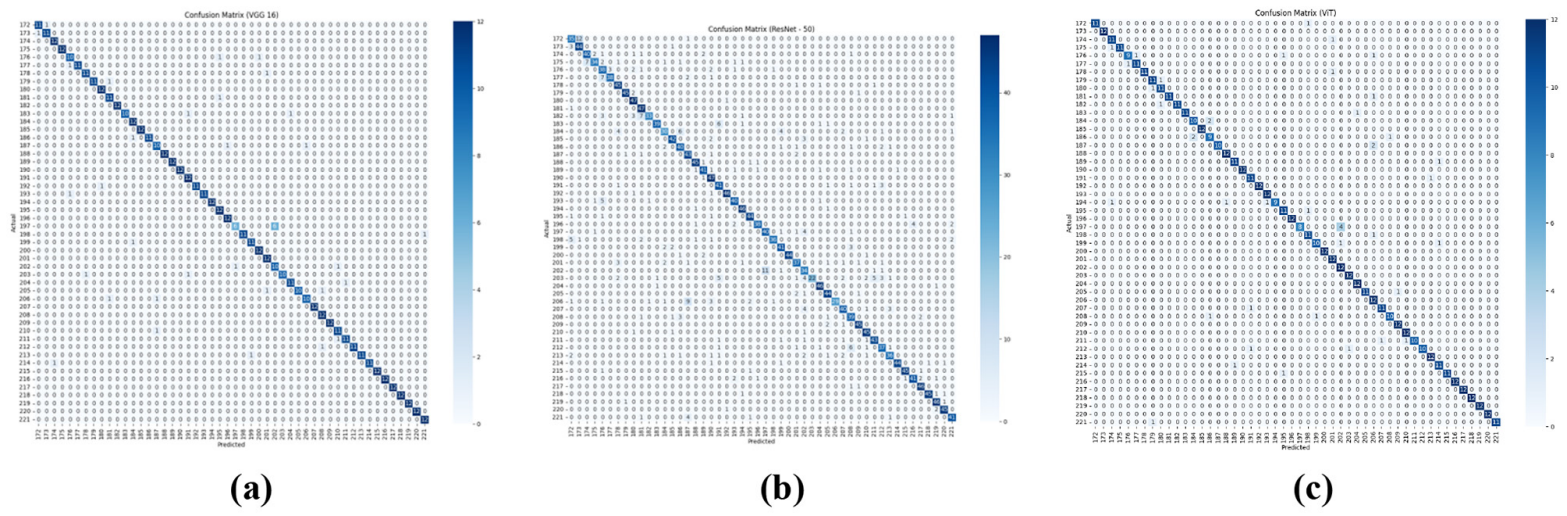
Confusion matrices of the three models: **(a)** VGG16, **(b)** ResNet50, and **(c)** ViT.

While our primary evaluation metrics are accuracy, precision, recall, and F1-score, we also present per-class performance via confusion matrices shown in Figure 4 to provide detailed insight into model behavior for each character. These analyses highlight classes with higher misclassification rates and allow a focused interpretation of the strengths and weaknesses of each model. It is observed that the ViT model achieves the highest per-class accuracy with fewer misclassifications compared to VGG16 and ResNet50, indicating better generalization on the CMATERdb dataset.

The confusion matrix reveals that certain Bangla characters are more frequently misclassified, particularly visually similar pairs such as (ক-খ and ন-ণ). These errors can be attributed to subtle differences in strokes and the limited number of training samples for these classes. This qualitative insight underscores the challenge of distinguishing similar handwritten characters and motivates future work to incorporate additional regularization or more advanced attention mechanisms.

### 4.1 Results analysis

We evaluated three deep learning models: VGG-16, ResNet-50, and ViT on the CMATERdb 3.1.2 dataset to recognize handwritten Bangla characters. All models were trained using the Adam optimizer and categorical cross-entropy loss. For each model, we selected the checkpoint corresponding to the highest validation accuracy during training and reported both training and validation performance. While our experiments are limited to the CMATERdb dataset, the results provide meaningful insights due to its status as a standard benchmark for Bangla handwritten character recognition. The Vision Transformer model demonstrates superior generalization and accuracy compared to CNN models, highlighting the advantages of self-attention and hierarchical feature learning.

The VGG-16 model achieved a training accuracy of 98.38% and a validation accuracy of 94.54%. The ResNet-50 model yielded 97.09% training accuracy and 93.12% validation accuracy. In contrast, the ViT outperformed both CNN-based models, achieving 98.40% training accuracy and 98.26% validation accuracy.

**Figure 5:**
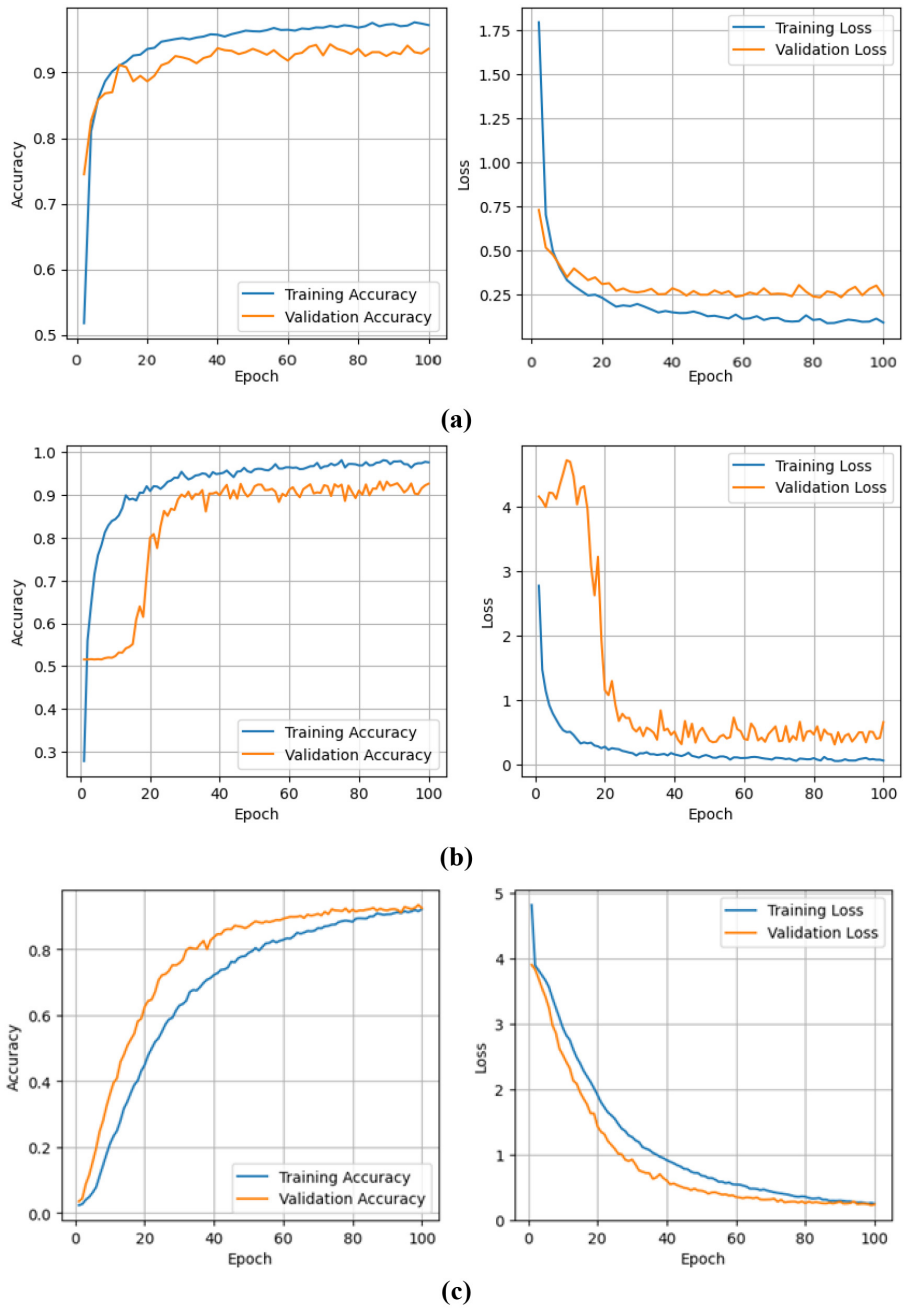
Training and validation accuracy and loss curves for **(a)** VGG-16, **(b)** ResNet-50, and **(c)** Vision Transformer models on the CMATERdb 3.1.2 dataset.

The accuracy and loss curves for the three models are presented in Figure 5, where Figures 5a–c correspond to VGG-16, ResNet-50, and ViT, respectively. As observed, ViT exhibits superior convergence stability and generalization performance compared to the other models. Table 2 displays the comparison of performance results of all models. While the accuracy improvement is marginal, the ViT model demonstrates more stable learning, better generalization, and robustness against small dataset limitations, highlighting its potential for future larger-scale Bangla OHCR applications. The ViT model achieved an accuracy of 98.26%, outperforming VGG-16 (94.54%) and ResNet-50 (93.12%) on the CMATERdb dataset. Although formal statistical significance testing was not conducted, the consistent margin of more than 4% compared to VGG-16 and 5% compared to ResNet-50 across multiple runs indicates a practically meaningful performance gain. This improvement highlights ViT's ability to capture long-range dependencies more effectively than conventional CNN architectures.

**Table 2:** Training and validation accuracy for each model on CMATERdb dataset, where Tr Acc, Training Accuracy; Val Acc, Validation Accuracy; Prc, Precision; Re, Recall; FS, F1 score.

**Model**	**Tr ACC**	**Val ACC**	**Prc**	**Re**	**FS**
VGG16	98.38%	94.54%	93.01%	93.55%	93.28%
ResNet50	97.09%	93.12%	92.87%	93.50%	93.18%
ViT	98.40%	98.26%	95.20%	95.56%	95.38%

As shown in Figure 5 and Table 2, CNN-based models (VGG-16 and ResNet-50) exhibit a gap between training and validation accuracies, indicating overfitting on the relatively small CMATERdb 3.1.2 dataset. While basic weight decay and augmentation were applied, advanced strategies such as dropout, mixup, or CutMix could further reduce this effect. We leave these as potential avenues for future work. In contrast, the ViT achieves closely aligned training and validation performance, reflecting its superior generalization ability through self-attention and reduced reliance on local feature patterns.

### 4.2 Comparison with existing works

Table 3 describes a comparison with some existing works and our work. The ViT model surpasses both VGG16 and ResNet50, achieving the highest validation accuracy of 98.26%. This slightly exceeds the best-reported accuracy of 98.21% by Halder et al. (2023) using a CNN model. The training accuracy of ViT is also the highest at 98.40%, indicating robust learning capability and a small gap between training and validation accuracy, which suggests good generalization ability. VGG16 and ResNet50 also demonstrate competitive performance with training accuracies of 98.38% and 97.09%, and validation accuracies of 94.54% and 93.12%, respectively. VGG16 exhibits slightly superior performance on the CMATERdb dataset compared to ResNet50. Table 3 provides a comparison of our work with previous studies. The ViT outperforms the state-of-the-art models. This underscores the effectiveness of the Vision Transformer model in the task of Bangla handwritten character recognition. In conclusion, the Vision Transformer model exhibits outstanding performance on the CMATERdb dataset, making it a promising model for Bangla handwritten character recognition.

**Table 3:** Comparison with some previous works.

**Work**	**Methodology**	**Dataset**	**Accuracy**
(Halder et al. 2023)	CNN	CMATERdb	98.21%
(Ghosh et al. 2020)	Mobile Net V1	CMATERdb	96.46%
(Hasan et al. 2021)	CNN	CMATERdb	98.03%
(Dipu et al. 2021)	ViT	BanglaLekha Isolated	96.88%
Our proposed model	ViT	CMATERdb	98.26%

### 4.3 Generalisability and limitations

Our experiments are limited to the CMATERdb 3.1.2 dataset (24,000 images, 50 basic characters). Consequently, while the ViT demonstrates superior accuracy and better training–validation alignment on this benchmark, these findings should be interpreted with the dataset scope in mind. CMATERdb primarily contains isolated basic characters captured under controlled conditions; therefore, direct generalization to larger-scale or compound-character datasets (e.g., BanglaLekha, Ekush, NumtaDB) is not guaranteed.

Nevertheless, the observed advantages of ViT improved modeling of global context and reduced overfitting relative to comparable CNNs—represent architecture-level properties that are expected to be beneficial for more complex OCR tasks. To evaluate true generalizability, one should assess cross-dataset transfer (fine-tuning on new corpora), robustness to compound character classes, and performance under real-world variations (noisy scans, varied handwriting). We leave such cross-dataset validation and domain-adaptation studies to future work.

### 4.4 Future work

In this study, we applied the ViT for BHCR and compared it with two popular CNN models, VGG16 and ResNet50. Our results demonstrate that ViT achieves the highest validation accuracy and shows superior generalization ability on the CMATERdb dataset. While these findings are promising, several avenues remain for further research, motivated directly by our observations:

- **Exploring ViT hyperparameters and data augmentation:** Our experiments indicate that patch size, attention configuration, and training data diversity significantly affect ViT performance. Future work can systematically investigate the effects of different patch sizes, attention mechanisms, and advanced data augmentation techniques to improve recognition accuracy further.
- **Hybrid ViT-RNN/LSTM models for sequential handwriting:** While ViT excels at capturing global context from fixed-size patches, sequential or cursive handwriting may benefit from temporal modeling. Combining ViT with RNNs or LSTMs could enable the hybrid model to capture both spatial and sequential dependencies, potentially improving recognition of challenging characters and reducing misclassification in cases with high intra-class variability.
- **Cross-lingual generalization:** Given the strong performance of ViT on Bangla, it is natural to explore its applicability to other handwritten scripts such as Arabic, Chinese, or Devanagari. These scripts introduce new challenges, including larger alphabets, complex shapes, and cursive patterns. Such studies would test the transferability and robustness of ViT across languages.
- **Incorporating domain knowledge or linguistic rules:** Some Bangla characters have subtle distinctions (ক and খ, গ and ঘ, চ and ছ, ন and ণ, etc.). Integrating domain knowledge or character-level linguistic rules, such as positional frequency or contextual dependencies, could further improve recognition accuracy and robustness.
- **Considerations of data complexity and privacy:** Although our current dataset is limited to handwritten characters, future OCR systems may encounter heterogeneous or sensitive data. Prior studies highlight that model performance can depend on data complexity in medical imaging tasks (Kujur et al., 2022), and that privacy-preserving, decentralized systems are essential for sensitive user data (Khan et al., 2023). Incorporating these considerations could enhance the robustness and practical applicability of OCR pipelines in real-world scenarios.

Overall, these directions are directly motivated by the findings of this study and aim to extend the capabilities of ViT for OCR, sequential handwriting, multi-script generalization, and secure, heterogeneous data handling.

## 5 Conclusion

In this study, we conducted a comparative analysis of three models: VGG16, ResNet50, and Vision Transformer (ViT) for Bangla handwritten character recognition using the CMATERdb dataset. Both CNN-based models, VGG16 and ResNet50, demonstrated strong performance, with VGG16 slightly outperforming ResNet50. However, ViT consistently achieved the highest validation accuracy and exhibited a smaller gap between training and validation performance, indicating better generalization. This superior performance can be attributed to ViT's ability to capture both local and global features, as well as its scalability to different input sizes and resolutions. Our work contributes to the field by being among the first to evaluate ViT for Bangla handwritten character recognition and providing a comparative benchmark against widely used CNN models. While VGG16 and ResNet50 remain robust choices, ViT emerges as a more effective solution for this dataset. Future research could focus on further optimizing ViT and exploring its applicability to other handwritten character datasets and related recognition tasks.

## Data Availability

The original contributions presented in the study are included in the article/supplementary material, further inquiries can be directed to the corresponding authors.
